# Sexualverhalten und Prävention sexuell übertragbarer Infektionen unter Berücksichtigung der SARS-CoV-2-Pandemie. Daten aus einem Versorgungszentrum für sexuelle Gesundheit und Medizin – WIR

**DOI:** 10.1007/s00103-021-03441-7

**Published:** 2021-10-19

**Authors:** Norbert H. Brockmeyer, Anja Potthoff, Wiltrud Knebel-Brockmeyer, Britta Köhler, Sandeep Nambiar, Janet Wach, Tobias Rodrigues Martins, Mona Uhrmacher, Ann-Kathrin Schuppe, Carsten Tiemann, Andre Kasper, Miriam Basilowski, Arne Kayser, Adriane Skaletz-Rorowski

**Affiliations:** 1 WIR – Walk In Ruhr, Zentrum für Sexuelle Gesundheit und Medizin, Große Beckstraße 12, 44787 Bochum, Deutschland; 2 Interdisziplinäre Immunologische Ambulanz, Zentrum für Sexuelle Gesundheit und Medizin, Klinik für Dermatologie, Venerologie und Allergologie, Ruhr-Universität Bochum, Bochum, Deutschland; 3 Gesundheitsamt der Stadt Bochum, Bochum, Deutschland; 4 Labor Krone/Labcon-OWL, Bad Salzuflen, Deutschland; 5 AIDS-Hilfe Bochum e. V., Bochum, Deutschland

**Keywords:** Sexuelle Gesundheit, Versorgungsforschung, Präventionsstrategien, Sexuelle Präferenzen, Online-HIV-/STI-Risikotest, Sexual health, Healthcare research, Prevention strategies, Sexual preferences, Online HIV/STI risk test

## Abstract

**Hintergrund:**

Anwendungsbezogene Daten zu sexueller Gesundheit und sexuellem Verhalten in unterschiedlich sexuell aktiven Populationen stehen nur begrenzt zur Verfügung, sind aber für Präventions- und Versorgungsstrategien sehr relevant. Das multisektorale Versorgungszentrum WIR – Walk In Ruhr hat aufgrund seiner Besucher*innenstruktur Zugang zu Daten aus diversen Lebenswelten.

**Ziel der Arbeit:**

Aus verschiedenen WIR-internen Datenquellen sollen populationsbezogene Erkenntnisse zu Alter, Geschlecht, sexueller Orientierung, Sexual- und Risikoverhalten gewonnen und Bedarfe für Prävention abgeleitet werden. Einflüsse der SARS-CoV-2-Pandemie auf das Sexualverhalten sollen durch den Vergleich verschiedener Zeiträume untersucht werden.

**Methoden:**

Ausgewertete Datenquellen sind der Onlinerisikotest für HIV und STI, die COWIR- und PrEP-Studie sowie die Immunologische Ambulanz und das Gesundheitsamt im WIR.

**Ergebnis:**

Sexuell übertragbare Infektionen (STI) sind von 2019 auf 2020 trotz Kontaktbeschränkungen gestiegen. Generell haben junge Menschen, Männer, die Sex mit Männern, und Frauen, die Sex mit Frauen haben, ein erhöhtes STI-Risiko aufgrund der gewählten Sexualpraktiken und der Anzahl sexueller Kontakte. Eine hohe Zahl bi- und transsexueller Kontakte ist festzustellen. SARS-CoV‑2 führte zu einer Reduzierung der Sexualkontakte. Sexualpraktiken wurden weiter gelebt. Die STI-Testquote und die Behandlungsrate stiegen an.

**Diskussion:**

Die Daten aus dem WIR belegen, dass eine junge Klientel mit aktivem Sexualleben erreicht wird. Die Ergebnisse aus Fragebögen und dem Onlinerisikotest zeigen den Zusammenhang von erhöhten positiven STI-Tests mit Sexualverhalten und sexuellen Präferenzen, weshalb spezifische Strategien zu Sexualaufklärung, Prävention, Tests und Therapien erforderlich sind.

## Abstract (en)

**Background:**

Application-based data regarding sexual health and sexual behavior in various sexually active populations are scarce but at the same time relevant with regards to prevention and healthcare supply strategies. Given the structure of its attendees, the Walk In Ruhr (WIR) Center for Sexual Health and Medicine is able to obtain data from diverse living environments.

**Objectives:**

Based on the online HIV/STI risk test, questionnaires, and attendee data from the WIR, this study aims to deduce population-related findings with regards to age, gender, sexual orientation, and sexual and risk behavior as well as the respective needs for prevention. The influence of the SARS-CoV-19 pandemic on sexual behavior is examined by comparing various phases.

**Methods:**

The analyzed data sources are the online HIV/STI risk test, the COWIR, and the PrEP study as well as the immunological outpatient clinic and the public health department at the WIR.

**Results:**

Notwithstanding contact restrictions, sexually transmitted infections (STIs) have increased from 2019 to 2020. Apart from men having sex with men and females having sex with females, young people also have an increased risk of STIs based on sexual practices and the number of sexual contacts. A large number of bisexual and transsexual contacts was found. SARS-CoV‑2 led to a decrease in sexual contacts; sexual practices continued. There was a growing proportion of STI tests and the treatment rate including partner treatment rose.

**Discussion:**

Data from the WIR center show that young attendees with an active sexual life are being reached. The results from questionnaires and the online HIV/STI risk test are mirrored in increased positive STI test results. These results vary depending on sexual behavior and sexual preferences such that specific strategies for sexual education, prevention, testing, and therapy are required.

## Einleitung

Sexuell übertragbare Infektionen (STI) mit *Treponema pallidum* (TP, Syphilis [1]), *Chlamydia trachomatis* (CT) und *Neisseria gonorrhoeae* (NG) nehmen in Deutschland und Europa konstant zu [2]. Antibiotikaresistenzen von NG und *Mycoplasma genitalium* (MG) sind eine Herausforderung für die Therapie [3], zu MG wird aktuell eine Leitlinie von der Arbeitsgemeinschaft der Wissenschaftlichen Medizinischen Fachgesellschaften e. V. (AWMF) erarbeitet. Bei HIV-Neuinfektionen war von 2018 auf 2019 eine leichte Zunahme um 8 % zu verzeichnen und von 2019 auf 2020 ein Rückgang um 21 % [4, 5].

Bestehende Präventionskampagnen scheinen nicht auszureichen, um sichtbare Rückgänge bei STI zu bewirken und die sexuelle Gesundheit der Bevölkerung nachhaltig zu verbessern. Entgegen den Erfolgen bei der HIV-Prävention konnte bisher weder das Wissen zu den häufigsten STI wie CT und humanen Papillomaviren (HPV) noch die Wahrnehmung von Präventionsmaßnahmen, wie z. B. CT-Test und HPV-Impfung, in Deutschland zufriedenstellend beeinflusst werden [6–9]. Sexuelle Gesundheit steht nicht für sich allein, sondern ist Bestandteil der Gesamtgesundheit und des Wohlbefindens einer Person und der Gesellschaft. Sexuelle, physische und psychische Erkrankungen bzw. Probleme können sich gegenseitig beeinflussen, sodass Kenntnisse über deren Wechselwirkungen für Beratung, Prävention, Diagnostik und Therapie notwendig sind [10, 11]. Die neue ärztliche Zusatzweiterbildung Sexualmedizin kann einen Beitrag leisten, diesbezüglich bestehende Defizite in der ärztlichen Ausbildung zu mindern [11–13]. Da STI ein wesentlicher und oft beeinträchtigender Faktor der sexuellen Gesundheit sind – von sexueller Dysfunktion über Schwangerschaftskomplikationen und Infertilität bis zu erhöhtem Tumorrisiko, z. B. durch HPV –, gilt es, durch Präventionsmaßnahmen die Prävalenz von STI zu senken [14–17]. Sexuelle Aktivität und Sexualpraktiken beeinflussen entscheidend das Risiko für eine STI ebenso wie die sexuelle Orientierung, Lebensalter, die Anzahl von Sexualpartner*innen und weitere Lifestyleaspekte, etwa übermäßiger Alkohol- und Drogengebrauch [18, 19]. Daher ist es unabdingbar, entsprechende Daten als Basis für Präventionsstrategien zur Verfügung zu haben.

Die in den letzten Jahren in den USA, der EU und anderen Ländern publizierten Studien beschäftigen sich mit sexueller Gesundheit und Sexualverhalten der Bevölkerung und einzelner Kollektive der Bevölkerung. Die EMIS-Studie (European MSM Internet Survey) zielt auf das Sexualverhalten und die Gesundheit schwuler und bisexueller Männer in Europa ab [20–22], Natsal (National Surveys of Sexual Attitudes and Lifestyles United Kingdom) legt den Fokus auf das Sexualverhalten des britischen Bevölkerungsquerschnitts und dessen Veränderungen über mehrere Jahrzehnte hinweg [18, 23]. Beide Studien liefern wertvolle Daten als Grundlage für die Erarbeitung bedarfsorientierter Strategien, indem sie aufzeigen, dass Einstellungen und verschiedene Lifestyles von Individuen für die Wahrnehmung von Präventionsangeboten von hoher Relevanz sind.

Mit der GeSiD-Studie (Sexualität und Gesundheit in der deutschen Gesamtpopulation) ist es erstmals gelungen, repräsentative Daten zum sexuellen Verhalten der Allgemeinbevölkerung in Deutschland wissenschaftlich zu erheben. Damit liegen bislang fehlende Daten zu Sexualverhalten, -praktiken, zu sexueller Aktivität, zu Wissen und Kommunikation zu STI u. v. m. vor und damit eine Grundlage, weitere Präventionsstrategien zu sexueller Gesundheit zu entwickeln [10, 11, 14, 24]. Versorgungsnahe, anwendungsbezogene Daten zum Sexualverhalten und zu sexuellen Praktiken sowie zu STI-Inzidenzen unterschiedlich sexuell aktiver Gruppen und ihrer jeweilig gelebten Sexualität gibt es in Deutschland bisher nur begrenzt.

Das WIR – Walk In Ruhr, Zentrum für Sexuelle Gesundheit und Medizin in Bochum, bietet aufgrund des multisektoralen, institutions- und rechtsformübergreifenden Konzeptes die Voraussetzungen für einrichtungsübergreifende, anwendungsbezogene Daten und Erkenntnisse, die aus der klinischen Arbeit abgeleitet werden können. Das WIR ist ein Zusammenschluss aus der interdisziplinären Immunologischen Ambulanz (IA) der Klinik für Dermatologie, Venerologie und Allergologie, Ruhr Universität Bochum, des Gesundheitsamtes (GA) Bochum, der Aidshilfe (AH) Bochum e. V. sowie Madonna e. V., ProFamilia e. V. und Rosa Strippe e. V. [25]. Ansprache und Präventionsbotschaften werden im WIR an unterschiedliche Populationen angepasst, sodass es gelingt, diese für sexuelle Gesundheit zu interessieren und für Prävention und Therapie zu gewinnen [7, 26]. Gleichzeitig werden für die jeweiligen Präventionsbotschaften Zugänge zu den Lebenswelten geschaffen, häufig in aufsuchender Arbeit durch sogenannte Health Adviser*innen (HA) – bspw. in Schulen, in Betrieben bis hin zu Aufklärungs- und Testangeboten in Swingerclubs. Damit erreicht das WIR eine diverse, sexuell aktive Klientel. Psychosoziale Versorgungsdaten der Aidshilfe aus aufsuchender Arbeit und vom WIR werden getrennt präsentiert. Die erhobenen Daten zu Soziodemografie, Geschlecht^1^, sexueller Orientierung, STI-Inzidenzen und Behandlungen liefern Erkenntnisse für bedarfsorientierte, zielgruppenspezifische Primär- und Sekundärprävention.

Die vom WIR erreichten Kollektive lassen sich in folgende 3 übergeordnete Populationen unterteilen, deren Daten auf unterschiedlichen Wegen gewonnen werden:

- junge, sexuell aktive Population mit Interesse an sexueller Gesundheit und Risikoeinschätzung. Es werden die Daten des Online-HIV-/STI-Risikotests (ORT) des WIR genutzt.
- Junge, sexuell aktive Population, mit aktuellem Bedarf für Beratung und Testung. Hierfür werden Daten aus der Beratungsstelle des Gesundheitsamtes im WIR (GA) herangezogen.
- Unterschiedliche Populationen mit vielfältigen sexuellen Erfahrungen und diversen sexuellen Vorlieben aus unterschiedlichen Lebenswelten – mit Einbindung in die medizinische Versorgung. Hierzu werden Daten der Immunologischen Ambulanz des WIR (IA) sowie der COWIR- und PrEP-Studie des WIR ausgewertet.

Zur sexuellen Versorgung während der SARS-CoV-2-Pandemie zeigen bisher vorliegende Studien unterschiedliche Ergebnisse. Es wird fast einheitlich über eine geringere Nutzung von STI-Test- und Beratungseinrichtungen sowie Arztkontakten berichtet [27–29], so sind die Ergebnisse zur Anzahl der Sexual- und Partner*innenkontakte sowie zu neu erworbenen STI teils kontrovers [30, 31]. Gleiches gilt für die Adhärenz in Bezug auf Gebote des Abstandhaltens (Physical Distancing) von Jugendlichen und jungen Erwachsenen [32]. Daten aus dem WIR (ORT, COWIR- und PrEP-Studie) dokumentieren die Veränderungen in der Pandemiezeit und liefern mögliche Erklärungsansätze, die als Grundlage für Präventionsangebote und Angebote zu sexueller Gesundheit in Pandemiezeiten dienen können.

In dem vorliegenden Beitrag werden zunächst die verschiedenen Datenquellen (ORT, COWIR-Studie, PrEP-Studie, IA, GA) vorgestellt und die angewandten Auswertungsmethoden beschrieben. Es folgt die Darstellung der jeweiligen Ergebnisse zu Soziodemografie, Geschlecht, sexueller Orientierung, STI-Inzidenzen und Behandlungen der WIR-Klientel sowie zu Veränderungen während der SARS-CoV-2-Pandemie. In der Diskussion werden die Erkenntnisse v. a. in Hinblick auf eine bedarfsorientierte, zielgruppenspezifische Primär- und Sekundärprävention diskutiert.

## Methoden

Das WIR – Walk In Ruhr, Zentrum für Sexuelle Gesundheit und Medizin, hat 2017 die Studie „Präventionsstrategie für HIV und STI des WIR“ initiiert (Ethikkommission der Medizinischen Fakultät der Ruhr-Universität Bochum, Zulassungsnummer: 17-6208). Verschiedene Instrumente, wie ORT, COWIR-Studie, PrEP-Studie sowie Auswertungen der IA- und GA-Daten sind darunter zusammengefasst und für die Teilnahme sind differenzierte Einverständniserklärungen notwendig.

Das Bindeglied aller Untersuchungen ist, Daten zu Wissen über Sexualität, zu sexuellen Präferenzen sowie Risikokonstellationen und Präventionsverhalten in unterschiedlichen Kollektiven zu generieren.

Zur Analyse der Veränderungen während der Pandemie werden bei den Auswertungen des ORT, der COWIR- und der PrEP-Studie die Daten von jeweils 2 Zeiträumen verglichen. Diese Zeiträume richten sich nach dem ersten Lockdown, der von seiner Wirkung auf Patient*innen einschneidende Folgen hatte und mit einer deutlichen Senkung der Besuche, insbesondere auch von PrEP-Nutzer*innen der HIV-Präexpositionsprophylaxe (PrEP), einherging. Ein weiteres Kriterium war, dass im WIR eine Notversorgung durchgeführt wurde (Notfallpatient*innen, wichtige Untersuchungen und Medikamentenverordnungen). Dies regulierte sich ab Mai 2020, sodass entsprechend das zweite Zeitintervall im ORT ab Mai gewählt wurde und ebenso die Studien COWIR und PrEP daran orientiert wurden.

### Online-HIV-/STI-Risikotest (ORT) des WIR.

Der anonyme ORT (www.risikotest.wir-ruhr.de/) ist eine webbasierte, prospektive Datenerfassung mit 26 Multiple-Choice-Fragen zu Soziodemografie, sexueller Orientierung, Sexualpraktiken, sexuellem Risikoverhalten, Impfung und STI-Lebenszeitprävalenz. Je nach Antwort öffnen sich spezifische Zusatzfragen. Die Testteilnehmer*innen erhalten die Möglichkeit, ihr eigenes HIV-/STI-Risiko einzuschätzen, und es werden an die Antworten angepasste Präventions- und Handlungsempfehlungen gegeben. Der Test schließt ab mit einer individuellen Risikoeinschätzung und weiterführenden Handlungsempfehlungen, von persönlichen Ermutigungen, Präventionsangebote zu nutzen, bis zu dem Rat, eine*n Arzt/Ärztin oder eine Beratungsstelle aufzusuchen. Zur Nutzung und Auswertung der ORT-Daten wird vor Testbeginn eine informierte Onlineeinwilligung des/der Nutzer*in eingeholt. Zum ORT wird breit informiert: Webseite des WIR, in Schulen, in Lifestyleclubs, in unterschiedlichen Flyern zu STI, bundesweit bei Fortbildungsveranstaltungen und durch Presseberichte. Zwischen 12/2017 und 04/2021 haben 33.561 Nutzer*innen den Test gestartet und 17.623 die Onlinebefragung vollständig durchgeführt. Nur Testteilnehmer*innen mit sexuellen Kontakten (16.550) wurden in die Auswertung eingeschlossen. Die Gesamtkohorte wurde in 2 zeitintervalldefinierte Kohorten geteilt: K1 mit 10.688 Personen, Erhebung 12/2017 bis 04/2020 (Zeit vor und zu Beginn der Coronakrise, inkl. des ersten Lockdowns) und K2 mit 5882 Personen, Erhebung 05/2020 bis 04/2021 (Zeitspanne nach dem ersten Lockdown). Fragen zur sexuellen Präferenz wurden in K1 von 10.551 und in K2 von 5793 Personen beantwortet. K1 und K2 werden miteinander verglichen und Teilergebnisse in der vorliegenden Publikation vorgestellt.

### COWIR-Studie des WIR.

In die noch rekrutierende retrospektive COWIR-Studie, die 02/2021 begann, sollen insgesamt 900 junge, kontaktfreudige, sexuell aktive Nutzer*innen des WIR eingeschlossen werden: 300 HIV-positive Patient*innen, 300 PrEP-Nutzer*innen sowie 300 HIV-negative, antiretroviral naive (AR-naive) Personen (keine PrEP oder HIV-Postexpositionsprophylaxe [PEP]). Die Proband*innen werden sowohl persönlich als auch virtuell (am Bildschirm) vom WIR über die Studie informiert. Die Rekrutierung erfolgt durch die Institutionen im WIR. Für die vorliegende Arbeit werden Teilmengen folgender in der COWIR-Studie genutzter Fragebögen verwendet: WIR-Erstbefragungsbogen (23 Fragen), COWIR-Fragebogen (11 Fragen), PrEP-Erstbefragung (18 Fragen). Neben soziodemografischen Daten werden PrEP-spezifische Daten sowie Daten zur Änderung des Sexual- und Risikoverhaltens vor dem ersten Lockdown (Pre‑L, Januar bis 22.03.2020) und ab Beginn des Lockdowns (Ex‑L; ab 23.03.2020) erhoben. Bisher (19.04.2021) wurden 515 Personen eingeschlossen, 9 wurden wegen teils fehlender Angaben in dieser Auswertung nicht berücksichtigt: 344 Männer, die Sex mit Männern haben (MSM), 87 heterosexuelle Männer, 57 heterosexuelle Frauen und 18 Frauen, die Sex mit Frauen haben (FSF). Die Antworten werden für die vorliegende Arbeit in einer Zwischenanalyse ausgewertet.

### PrEP-Studie des WIR.

Die PrEP-Studie ist eine monozentrische, prospektive Beobachtungsstudie, die 2018 begonnen wurde und allen PrEP-Nutzer*innen (≥18 Jahren) des WIR angeboten wird [33]. Im Rahmen dieser Publikation werden Teilergebnisse von 138 Teilnehmer*innen vorgestellt, die nach dem ersten Lockdown ab Mai 2020 bis 30.04.2021 das WIR wieder aufsuchten, um die PrEP-Einnahme fortzusetzen, und ergänzend zur PrEP-Erstbefragung die Zusatzfrage „Änderung der Zahl der Sexualpartner*innen in der Pandemie“ nach persönlicher Ansprache beantworteten.

### Daten aus der Immunologischen Ambulanz (IA) des WIR.

Anonymisierte Patient*innendaten aus der IA von 6300 Patient*innen aus den Jahren 2019 und 2020 und einige Vergleichsdaten aus dem Jahr 2016 werden in diese Publikation einbezogen, zum einen soziodemografische Daten, zum anderen klinische Daten zu Inzidenzen und Behandlung von HIV und anderen STI.

### Daten aus dem Gesundheitsamt (GA) im WIR.

Aus dem GA wurden anonymisierte Daten von 4200 Klient*innen aus den Jahren 2019 und 2020 – soziodemografische Daten sowie Daten zu STI, Geschlechterverteilung und Behandlungsquote – in die Arbeit eingeschlossen.

Die Auswertung aller Daten erfolgte mittels deskriptiver Statistik. Die Proband*innencharakteristika der Studienpopulationen wurden durch Häufigkeitstabellen, Mittelwerte oder Mediane und Quartile deskriptiv ausgewertet.

## Ergebnisse

### Online-HIV-/STI-Risikotest des WIR (ORT)

Zunächst werden Basisdaten des ORT von 12/2017 bis 04/2021 vorgestellt, unabhängig von einer Differenzierung in Kohorte 1 und 2. Der ORT erreichte mit monatlich 400–500 Nutzer*innen eine eher junge Zielgruppe (84 % sind 18- bis 39-Jährige), mit einem Anteil von jeweils rund 40 % heterosexueller Frauen/Männer (Tab. 1). Über 25 % der MSM und über 80 % der FSF sind bisexuell. Trans Personen nutzten zu 0,6 % den ORT (nicht ausgewertet wegen kleiner Fallzahl). Während rund 40 % im ORT eine Sexualpartnerschaft pro Jahr angaben, nannte ca. ein Drittel 2–3 und etwa ein Viertel mehr als 3 Partner*innen pro Jahr. Beim Sexualverhalten gaben rund 70 % kondomlosen Sex und rund 30 % Analverkehr an. Etwa 3 % der Heterosexuellen und 8 % der MSM nannten Chemsex^2^.

**Table 1:** 

Angaben der Teilnehmenden	Kohorte 1 (K1)	Kohorte 2 (K2)
	(Dez. 2017–Apr. 2020)	(Mai 2020–Apr. 2021)
	(*n* = 10.668)^a^	(*n* = 5882)^a^
*Alter (Jahre)*		
<18	801 (7,5 %)	306 (5,2 %)
18–24	5171 (48,5 %)	2205 (37,5 %)
25–39	3836 (36,0 %)	2690 (45,7 %)
40–59	807 (7,6 %)	648 (11,0 %)
≥60	53 (0,5 %)	33 (0,6 %)
*Geschlecht*		
Weiblich	4938 (46,3 %)	2819 (47,9 %)
Männlich	5667 (53,1 %)	3029 (51,5 %)
Trans	63 (0,6 %)	34 (0,6 %)
*Sexuelle Orientierung*		
Hetero (♀)	4401 (41,7 %)^b^	2579 (44,5 %)^b^
Hetero (♂)	4297 (40,7 %)^b^	2036 (35,1 %)^b^
FSF^c^	536 (5,1 %)^b^	240 (4,1 %)^b^
Davon FSF bisexuell	451 (84,1 %)	209 (87,1 %)
MSM^d^	1317 (12,5 %)^b^	938 (16,2 %)^b^
Davon MSM bisexuell	424 (32,2 %)	229 (24,4 %)
*Sexualpartner pro Jahr*		
Nur 1	4750 (44,5 %)	2224 (37,8 %)
2–3	3257 (30,5 %)	2135 (36,3 %)
>3	2661 (24,9 %)	1523 (25,9 %)
*Sexualverhalten*		
Sex ohne Kondom	7232 (67,8 %)	4166 (70,8 %)
Analverkehr	3258 (30,5 %)	1830 (31,1 %)
Chemsex (chemische Drogen)	590 (5,5 %)	268 (4,6 %)
PrEP-Nutzer^e^	41 (0,4 %)	45 (0,8 %)
*HIV/STI*^f^*-Test*		
HIV-Test letzte 6 Monate	1267 (11,9 %)	805 (13,7 %)
HIV-positiv (in Kohorte)	16 (1,3 %)	9 (1,1 %)
HIV nie getestet	6497 (60,9 %)	3067 (52,1 %)
STI-Test letzte 6 Monate	1261 (11,8 %)	797 (13,5 %)
STI-Test positiv (jemals)	1106 (10,4 %)	821 (14,0 %)
STI nie getestet	7044 (66,0 %)	3350 (57,0 %)
*Behandlung*^g^		
Nur Patient	371 (33,5 %)	251 (30,6 %)
Patient und Partner	666 (60,2 %)	542 (66,0 %)
Test of Cure	735 (66,5 %)	579 (70,5 %)
Behandlungsquote	93,7 %	96,5 %

^a^Anzahl der Nutzer pro Monat in K1 = 361, in K2 = 507

^b^*n* = 10.551 in K1, *n* = 5793 in K2

^c^Frauen, die Sex mit Frauen haben

^d^Männer, die Sex mit Männern haben

^e^HIV-Präexpositionsprophylaxe (PrEP)

^f^STI wie Syphilis, Chlamydien, Gonorrhö

^g^Prozentsatz relativ zu den STI-positiven Personen (K1 *n* = 1106, K2 *n* = 821)

Einen HIV-Test in den letzten 12 Monaten gaben über 20 % der Heterosexuellen und 38 % der FSF und MSM an. Ein Test auf andere STI wurde von ca. 28 % der heterosexuellen Frauen, der MSM und FSF genannt sowie von 13 % der heterosexuellen Männer. „Noch nie“ wurden entsprechende Tests von rund 60 % der ORT-Nutzer*innen durchgeführt. HIV-positiv zu sein, wurde von ca. 1 % der MSM und von 2 heterosexuellen Männern (0,003 %) angegeben. Jemals positiv auf eine andere STI getestet worden zu sein, bejahten 10–14 % der Heterosexuellen und ca. 45 % der MSM. Die STI-Behandlungsrate lag bei etwa 95 %, Partner*innen wurden zu über 60 % mitbehandelt (Tab. 1).

Im Folgenden werden ausgewählte deutliche Veränderungen zwischen den beiden Kohorten (K1 von 12/2017 bis 04/2020 und K2 von 05/2020 bis 04/2021) dargestellt. Die Nutzung des anonymen ORT stieg in K2 (05/2020 bis 04/2021) um 40,4 % von monatlich 361 auf 507 Nutzer*innen an (Tab. 1). Die Quote der 18- bis 24-jährigen Testnutzer*innen blieb dabei absolut gesehen gleich, verringerte sich prozentual jedoch deutlich um 11 %, wohingegen sie bei den 25- bis 39-Jährigen um 9,7 % anstieg. Während den ORT 18- bis 24-jährige heterosexuelle Frauen/Männer weniger nutzten (12,9/12,1 %), stieg der Anteil der 25- bis 39-jährigen heterosexuellen Frauen/Männer um 12,8 %/10,2 %. Der Anteil der MSM stieg in K2 um 3,7 % auf 16,2 % (Tab. 1).

Bei den Sexualpraktiken zeigten sich kaum Änderungen, jedoch bei der Anzahl der Sexualpartner*innen. Der Anteil der Personen, die angaben, in einem Jahr 2–3, respektive >3 Partner*innen zu haben, stieg insgesamt in K2 um 6,8 % (Tab. 1), am meisten bei heterosexuellen Frauen und Männern mit 7,5 %/7,9 % und jungen Menschen (18–24 Jahre) mit 8,6 % (Tab. 2). Deutlich gesunken ist die Quote der bisexuellen Kontakte von MSM um fast 8 % auf 24,4 %, bei sonst unveränderter Anzahl von Partner*innen.

**Table 2:** 

Angaben der Teilnehmenden	Online-HIV-/STI-Risikotest								COWIR-Studie
	Kohorte 1	Kohorte 2	Kohorte 1	Kohorte 2	Kohorte 1	Kohorte 2	Kohorte 1	Kohorte 2	
	*N* = 801 (7,5 %)	*N* = 306 (5,2 %)	*N* = 5171 (48,5 %)	*N* = 2205 (37,5 %)	*N* = 3836 (36 %)	*N* = 2690 (45,7 %)	*N* = 807 (%)	*N* = 648 (11 %)	*N* = 105 (20,4 %)
	<18 Jahre	<18 Jahre	18–24 Jahre	18–24 Jahre	25–39 Jahre	25–39 Jahre	40–59Jahre	40–59 Jahre	<28 Jahre
*2–3 Sexualpartner*^a^	171 (21,3 %)	87 (28,4 %)	1490 (28,8 %)	763 (34,6 %)	1298 (33,8 %)	1046 (38,9 %)	285 (35,3 %)	230 (35,5 %)	–
*Mehr als 3 Sexualpartner*^b^	145 (18,2 %)	29 (9,5 %)	1077 (20,8 %)	520 (23,6 %)	1109 (28,9 %)	747 (27,8 %)	307 (38,0 %)	2016 (33,3 %)	93 (98,5 %)
*Sex ohne Kondom*	517 (64,5 %)	223 (72,9 %)	3644 (70,5 %)	1613 (73,2 %)	2553 (66,6 %)	1880 (69,9 %)	480 (59,5 %)	428 (66,0 %)	76 (72,2 %)
*Analverkehr*	209 (26,1 %)	70 (22,9 %)	1407 (27,2 %)	583 (26,4 %)	1307 (34,1 %)	898 (33,4 %)	309 (38,3 %)	269 (41,5 %)	41 (39,1 %)
*Chemsex (chemische Drogen)*	76 (9,5 %)	18 (5,9 %)	296 (5,7 %)	98 (4,4 %)	173 (4,5 %)	118 (4,4 %)	33 (4,1 %)	29 (4,5 %)	21 (20,4 %)
*Fisting (Faustverkehr)*	156 (19,5 %)	61 (19,9 %)	411 (7,9 %)	184 (8,3 %)	272 (7,1 %)	204 (7,6 %)	97 (12,0 %)	87 (13,4 %)	–
*HIV-Test nie getestet*	742 (92,6 %)	284 (92,8 %)	3825 (74,0 %)	1504 (68,2 %)	1707 (44,5 %)	1087 (40,4 %)	196 (24,3 %)	178 (27,5 %)	–
*STI-Test nie getestet*	708 (88,4 %)	260 (85,0 %)	3725 (72,0 %)	1429 (64,8 %)	2157 (56,2 %)	1329 (49,4 %)	419 (51,9 %)	314 (48,5 %)	–
*Lebenszeitprävalenz STI*	37 (3,4 %)	14 (4,5 %)	375 (7,2 %)	250 (11,4 %)	545 (14,2 %)	424 (15,7 %)	143 (17,7 %)	124 (19,1 %)	43 (40,8 %)
*Beschwerden im Intimbereich*	243 (30,3 %)	121 (39,5 %)	1061 (20,5 %)	597 (27,1 %)	722 (18,8 %)	561 (20,9 %)	148 (18,3 %)	107 (16,5 %)	–

^a^In Kohorte 1 und 2 innerhalb der letzten 12 Monate

^b^In der COWIR-Studie innerhalb der letzten 6 Monate

Beim Vergleich des Testverhaltens und der STI-Rate von K1 mit K2 fällt auf, dass die STI-Testquote anstieg, bei gleichzeitiger Zunahme der positiven STI-Testergebnisse (Tab. 1) und Zunahme von aktuellen Beschwerden im Intimbereich (Tab. 2). In den letzten 12 Monaten hatten sich 18- bis 24-Jährige mit rund 4 % häufiger testen lassen, ihre Lebenszeitprävalenz für STI stieg um 4,2 % und parallel nahmen ihre Angaben zu aktuellen Beschwerden im Intimbereich um 6,6 % zu (Tab. 2).

Insgesamt steigt die Quote der Partner*innen-Mitbehandlung um fast 6 % auf 66,0 % in K2, die des Tests zur Therapiekontrolle (Test of Cure) um 4 % auf 70,5 % (Tab. 1).

### COWIR-Studie des WIR

Im Zeitraum von Februar 2021 bis 19.05.2021 waren von 506 Teilnehmer*innen der COWIR-Studie 83,8 % Männer und 15,2 % Frauen. Davon waren 144 heterosexuelle Männer/Frauen (16,9 %/11,1 %), 344 MSM (66,9 %, davon 7,5 % bisexuell) und 18 FSF (3,5 %, davon 61,1 % bisexuell). HIV-positiv waren 33,0 %, die PrEP nutzten 32,5 %, und 34,5 % der Teilnehmer*innen waren AR-naiv. 105 Personen (20,4 %) waren jünger als 28 Jahre (Tab. 2). Über 95 % der Proband*innen hatten eine Schulbildung von mehr als 10 Jahren, Migrationshintergrund etwa 26 %.

**Figure 1:**
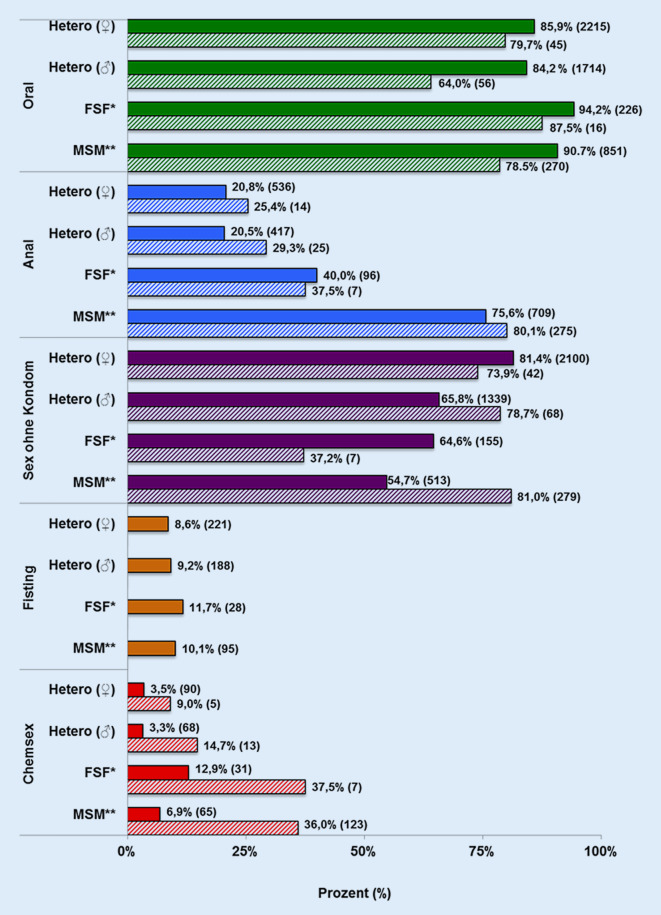

In der COWIR-Studie gaben Heterosexuelle für die letzten 6 Monate rund 3 Sexualkontakte an, FSF 4 und MSM 6 Partner*innen. 50,3 % der Proband*innen reduzierten ihre sexuellen Kontakte bereits in Pre‑L, in Ex‑L sogar 65,3 %. Durchschnittlich 80,8 % der Kohorte hatten Sex ohne Kondom, 36,0 % der MSM nutzten Chemsex, teils mit Alkohol, heterosexuelle Männer/Frauen zu 14,7 %/9,0 % (Abb. 1). MSM hatten die höchste Lebenszeitprävalenz von STI mit 75,3 %, Heterosexuelle mit rund 30,0 %. Jugendliche <28 Jahre gaben zu 40,8 % an, jemals eine STI gehabt zu haben (Tab. 2).

### PrEP-Studie des WIR

In die *PrEP-Studie* wurden im Zeitraum von Mai 2020 bis 30.04.2021 133 MSM und 5 Trans-Personen eingeschlossen. 26,1 % der Teilnehmer*innen waren jünger als 28 Jahre, der Altersdurchschnitt lag bei 32 Jahren (Spannweite 18–64 Jahre). Über 95 % hatten eine Schulbildung von mehr als 10 Jahren, Migrationshintergrund etwa 30 %.

In der PrEP-Studie reduzierten die Teilnehmer*innen ihre Sexualkontakte pandemiebedingt noch deutlicher: 63,8 % in Pre‑L und 80 % in Ex‑L geben weniger Sexualkontakte an. Sex mit unbekannten Partner*innen nannten 15,8 % in Ex‑L. Die durchschnittliche Partner*innenzahl in 6 Monaten reduzierte sich von 13 (bei einer Spannweite von 0–100) in Pre‑L auf 8 (bei einer Spannweite von 0–50) in Ex‑L.

### Immunologische Ambulanz (IA) des WIR

Die IA des WIR versorgte in den Jahren 2019 und 2020 ungefähr 2000 Patient*innen im Quartal, 18 % Frauen, 84 % Männer, <1 % Trans-Personen. Das mediane Alter der Patient*innen der IA ist 50 Jahre, rund ein Viertel ist 18–39 Jahre alt, 40–59 Jahre rund 60,0 % und älter als 60 Jahre sind ca. 15 %. HIV-positiv sind 45,7 % der Patient*innen, mit einem Altersdurchschnitt von 50,5 Jahren. Rund 500 PrEP-Nutzer*innen werden im WIR von der Aidshilfe und der IA betreut.

**Figure 2:**
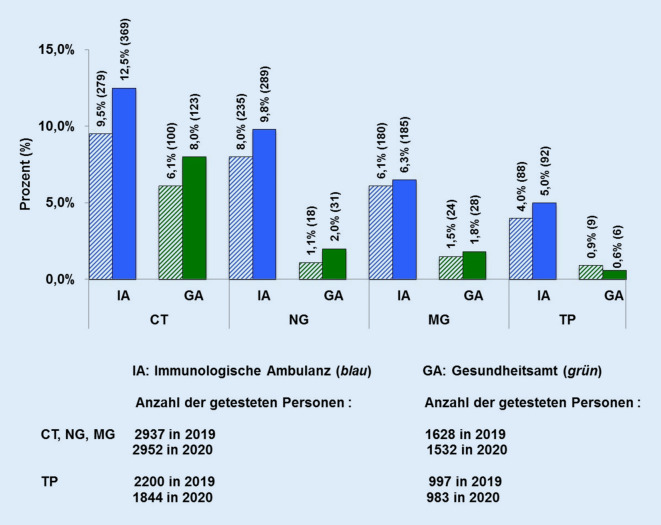

Die IA des WIR verzeichnete, verglichen mit 2019, im Pandemiejahr 2020 einen Anstieg der Patient*innen von 13 %. Die STI-Inzidenzen von CT, NG und MG nahmen im Pandemiejahr 2020 insgesamt um 149 Fälle (21,5 %) auf 843 Fälle (28,6 %) zu, verglichen mit 2019 (Abb. 2), bei einer gleich hohen STI-Testquote.

Bei den 18- bis 29-Jährigen waren 2020 die Infektionen mit CT (17,9 %), NG (11,4 %) und MG (7,6 %) am häufigsten. Um ein Drittel stiegen 2020 die NG-Infektionen bei den 30- bis 49-Jährigen auf 11,4 %. Seit einigen Jahren sind Änderungen in den Primärlokalisationen der STI zu beobachten: Die genitalen Infektionen mit CT und MG verdoppelten sich seit 2016 auf 52,6 % (2020). Die rektalen NG-Infektionen hingegen nahmen seit 2016 von 34,1 % auf 42,1 % zu. Bei MSM sind rund 55 % der STI rektal lokalisiert. NG wurden mit rund 30 % am häufigsten oral diagnostiziert.

Die HIV-Neudiagnosen sind seit 3 Jahren kontinuierlich auf zuletzt 8 festgestellte Infektionen im Jahr 2020 gesunken (bei einem noch 80 %igen Anstieg von 2016 auf 2017 von 17 auf 31 Personen), wohingegen die Quote der Syphilisneudiagnosen von 2016 bis 2020 kontinuierlich um 35,3 % auf 92 Personen gestiegen ist. Dies entspricht mehr als 10 % aller Neudiagnosen im Ruhrgebiet mit 5,1 Mio. Einwohnern (Vergleich der Infektionszahlen für CT, NG, MG und TP 2019/2020 in Abb. 2).

Von den in der IA behandelten Patient*innen wurde bei fast 90 % ein Test of Cure durchgeführt. Die Erfolgsquote schwankte, abhängig vom Erreger, zwischen 85–95 %, dabei waren die Ergebnisse für MG am schlechtesten.

### Gesundheitsamt (GA) im WIR

Die Beratungsstelle für sexuelle Gesundheit hat in den Jahren 2019/2020 zwischen 2000 und 2300 Klient*innen im Jahr beraten. Die Geschlechterverteilung bei Heterosexuellen war fast gleich. 70 % der Klient*innen waren zwischen 20 und 29 Jahre alt, 20 % zwischen 30 und 39 Jahre. 70 bis 80 % der Klient*innen wurden auf STI (CT, NG, MG) getestet.

Die Zahl der Klient*innen des GA nahm im Jahr 2020 um 22,5 % auf 1821 ab, verglichen mit 2019. Die Inzidenz von CT, NG, MG stieg um 28,2 % auf 182 im Jahr 2020 an (CT um 23,0 %, MG um 16,7 % und NG um 72,2 %; Abb. 2). Getestet wurden 671 Frauen, 861 Männer, davon 300 MSM (ein Anstieg um 17,3 % verglichen mit 2019). Die Geschlechterverteilung Frauen/Männer war im Jahr 2020 bei CT (63/60) fast gleich. Die Zahlen positiver HIV-Tests und Syphilisneudiagnosen war im Jahr 2020 rückläufig (HIV auf 2 und TP auf 6 Fälle). Die Monatsstatistiken für das Pandemiejahr 2020 zeigen, dass in den kritischen Monaten der Pandemie die Zahl der Tests deutlich abnahm, die Zahl der positiven Tests aber parallel deutlich anstieg. Während im Januar 2020 von 204 Tests 11,3 % positiv waren, fiel die Zahl der Tests im März auf 127, davon 13,4 % positiv, im Mai waren von 49 Tests 28,6 % positiv. In den Sommermonaten stabilisierten sich die Zahlen (Juli: 168 Tests, davon 9,5 % positiv, August: 159, davon 6,3 % positiv). In der zweiten Pandemiewelle sanken die Testzahlen erneut auf 114 im November, davon 13,2 % positiv, und 125 im Dezember mit 14,4 % positiven Testergebnissen.

## Diskussion

Die Ergebnisse der Arbeit zeichnen ein inhomogenes Bild. Sie zeigen, dass unterschiedliche Zielgruppen und deren Sozialisierung in diversen Lebenswelten sowie sexuelle Präferenz, Partner*innenzahl, sexuelle Aktivität und Sexualpraktiken zu unterschiedlich hohen Risikokonstellationen für STI führen, was sich mit anderen Studien deckt [6, 8, 34, 35]. So nutzen bspw. junge Menschen bis 24 Jahre seltener ein Kondom als ältere, MSM nutzen bei einer höheren, teils sehr hohen Anzahl von Partner*innen häufiger chemische Drogen zum Sex. Ein mit 9 % in den Populationen gleichmäßig verteilter Risikofaktor ist Fisting^3^, das je nach Partner*innenzahl zu einer sehr hohen Infektionsrate von STI führen kann [36]. Um diese zu minimieren, bedarf es individueller Prävention und Bildung.

Die mit 71 % hohe Nutzung des ORT von jungen, bis 39 Jahre alten, heterosexuellen Menschen zeigt den Bedarf und das Interesse Jugendlicher und junger Erwachsener an Wissen zu Sexualität und STI sowie an der Einschätzung ihres Sexualverhaltens und damit verbundener Gesundheitsrisiken. Der Bedarf an Wissen zu Sexualität und STI wird in der GeSiD-Studie detailliert beschrieben und auch in der PreYoungGo-Studie bestätigt [6, 8]. Auch im GA sind 90 % der Klient*innen zwischen 20 und 39 Jahre alt, diese möchten beraten und häufig auch auf eine STI getestet werden.

Das Sexualverhalten Jugendlicher und junger Erwachsener zeigt, dass ein breites Spektrum an Sexualpraktiken wie Analverkehr^4^, Chemsex^5^ und Fisting kombiniert mit einer höheren Anzahl von Sexualpartner*innen gelebt wird, dies bei geringer (<30 % ORT/COWIR) Kondomnutzung. Da sich diese Populationen aber auch zu ca. 20 % (Daten des ORT) testen lassen (in den letzten 6–12 Monaten), scheint ein Basiswissen zu Risikofaktoren für die sexuelle Gesundheit vorhanden zu sein. Zudem nutzen sie zahlreich die Angebote bspw. des anonymen ORT, der anonymen Testung und Beratung des GA, der Aidshilfe und die vielfältigen Beratungs- und Testangebote durch HA im WIR. Verstärkt müssen <18-Jährige angesprochen werden, da sie trotz vielfältiger Risikokontakte und Sexualpraktiken zu 85 % noch nie einen STI-Test gemacht haben (Tab. 2). Die Botschaft für diese sehr jungen Heranwachsenden muss auch sein, dass ein eigenverantwortliches Handeln in Hinblick auf ihre sexuelle Gesundheit erforderlich ist.

Durch die aufsuchende Arbeit der HA ist es gelungen, Präventionsbotschaften und Testangebote auch gezielt in verschiedenen Lebenswelten zu verbreiten, z. B. in Schulen, bei Menschen mit Migrationshintergrund, in Swingerclubs und an anderen Orten sexuellen Lifestyles etc. [8]. Am Beispiel der Swingerclub-Nutzer*innen wird deutlich, dass bestimmte Lebenswelten mit höheren STI-Risiken verbunden sind und deshalb die direkte Ansprache (auch mit Testangebot vor Ort) notwendig ist. Wie wichtig es ist, passgenaue Botschaften und Testangebote in die jeweiligen Zielgruppen zu tragen, zeigen die in allen beschriebenen Populationen durchgängig hohen STI-Zahlen (wie CT, MG, NG) verglichen mit den Daten der GeSiD-Studie in der jungen Allgemeinbevölkerung [6] (Tab. 2).

Einen Überblick zu STI-Inzidenzen einer sexuell aktiven Zielgruppe mit medizinischem Versorgungswunsch geben die Daten^6^ der in der IA betreuten Patient*innen. Diese kommen aus einer breiten – über Bochum weit hinausreichenden – Industrie‑, Hochschul- und ländlichen Bevölkerung mit einem Altersmedian von 50 Jahren. Infektionen mit CT/NG/MG stiegen um 21,5 %, im GA um 28,2 %, einer mit dem ORT gut vergleichbaren Kohorte, an. Infektionen mit TP stiegen von 2019 auf 2020 in der IA um 4,6 % an. Wie auch in den anderen Populationen in ORT und COWIR ist dieser hohe Anstieg gerade auch bei jungen Menschen von 18–29 Jahren zu beobachten. Gleiches wird auch in anderen Kohorten beschrieben [31, 37, 38]. Die Mehrzahl der TP-Infektionen tritt jedoch bei ca. 10 Jahre Älteren auf [39]. Die STI-Zahlen der IA und des GA nahmen nicht nur relativ, sondern auch absolut zu, entsprechend einer realen Steigerung der STI in diesen beiden Kohorten – dies bei gleicher Symptomrate. Im ORT verläuft in K2 die Steigerung der Beschwerden im Intimbereich parallel zu der der STI.

Die in allen Kohorten hohen Raten an symptomlosen und steigenden STI-Inzidenzen weisen auf die Wichtigkeit risikoadaptierter STI-Testungen hin. Geringere Kondomnutzung, geänderte Sexualpraktiken, teils mit Chemsex, können einen Erklärungsansatz liefern [40–42]. Bei der Geschlechterverteilung der STI fällt auf, dass CT- und MG-Infektionen bei Männern und Frauen in der IA und dem GA im Jahr 2020 gleich häufig waren. CT hat in Deutschland eine rund zehnfach höhere Prävalenz als NG. Im WIR erhöhten sich die NG-Infektionen auch bei Frauen insgesamt und waren zu 30 % oral lokalisiert – eine Lokalisation, von der aus Infektionen wie CT oder NG leichter verbreitet werden können. Wie weit dies Bedeutung für einen weiteren Anstieg bei Frauen und Heterosexuellen insgesamt hat, muss beobachtet werden. Die Daten der IA weisen darauf hin, dass STI-Abstriche aus 3 Lokalisationen – oral, anal, genital – erfolgen sollten, da anderenfalls bis zu zwei Drittel der Infektionen nicht diagnostiziert werden. Da immer mehr Sexualkontakte in der Gesamtpopulation anal und oral, letztere fast immer ohne Kondom, stattfinden, ist für die Gesamtbevölkerung Aufklärung notwendig [6, 42–44].

Positiv ist in beiden Kohorten des ORT und der IA festzustellen, dass Therapie, Partner*innenmitbehandlung und Test of Cure verstärkt wahrgenommen werden (Tab. 1). Jedoch werden Präventionsmöglichkeiten durch Impfungen zu wenig erkannt [9] oder die Wege dahin sind nicht deutlich genug aufgezeigt (bspw. Hepatitis-B(HBV)-Impfrate bei MSM ca. 63 %, HPV ca. 26 % insgesamt und ca. 41 % bei Frauen im ORT). Enttäuschend ist die geringe HPV-Impfquote bei unter 24-Jährigen mit 37 %. Kampagnen zu Impfungen für Risikopopulationen, über HBV- und HPV-induzierte Erkrankungen und der Nutzen einer Impfung müssen neu formuliert und in ein breiteres Netzwerk der Ansprache eingebunden werden (z. B. über Schulen, Ärzt*innen, Vereine). Auch die Nutzung der PrEP ist noch nicht ausreichend in Präventionsbotschaften, insbesondere für Frauen umgesetzt [45]. Dazu passend gab keine Frau im ORT eine PrEP-Nutzung an, im WIR waren es 2 Frauen.

Menschen aus Lebenswelten mit sehr hohen Risikokonstellationen (MSM, FSF) scheinen sich dieser mehr bewusst zu sein und mehr Kenntnisse dazu zu haben [19, 46, 47]. Risikobehaftete Sexualpraktiken werden mit vielen Partner*innen gelebt, gleichzeitig werden Mechanismen wie PrEP (16.000 in Deutschland, 500 im WIR), häufige HIV- und STI-Tests (im ORT HIV-Tests ca. 60 % und andere STI-Tests ca. 50 %), Einbindung in Versorgungsangebote (im WIR bspw. rund 60 % MSM) als Sicherheit genutzt. Diese reichen jedoch nicht aus, um das höhere Infektionsrisiko auszugleichen [6, 8, 10, 48]. Wegen des hohen Anteils bisexueller Kontakte bei MSM von 25–30 % und FSF von 87 % im ORT (Tab. 1) und 7,5 % und 61,1 % in COWIR erscheint es notwendig, noch besser über die damit verbundenen Risiken aufzuklären und über Schutzmöglichkeiten zu informieren. Menschen mit sexuellen Präferenzen zu unterschiedlichen Populationen haben nicht nur höhere STI-Risiken aufgrund ihrer erhöhten und diversen Sexualpartner*innenkontakte sowie eine höhere STI-Rate, sondern auch ein breiteres Erregerspektrum für STI, weil in unterschiedlichen Populationen die Prävalenz bspw. von NG oder TP sehr hoch ist [49–52]. Dieser Zusammenhang von sexueller Orientierung und populationsspezifischen STI-Raten wird von Saxton et al. sehr gut analysiert und insofern ist die epidemiologische Bedeutung von populationsübergreifenden Sexualkontakten offensichtlich (Populationsswitch; [53]).

Wesentlich für den starken Rückgang der HIV-Infektionen in Deutschland im Jahr 2020 (wie auch seit 2017 im WIR) dürfte die erfolgreiche HIV-Therapie (TasP – Treatment as Prevention) sein. Dabei gelang es bei 94 % der Infizierten (im WIR 98 %) unter der HIV-Nachweisgrenze zu bleiben (*n* = *n*)^7^. Als weiterer wichtiger Grund kommt insbesondere die eingeschränkte internationale Mobilität hinzu. Ob sich in diesem Rückgang die Wirkung der PrEP bereits statistisch ablesen lässt – wie dies in anderen Ländern der Fall ist –, muss weiter beobachtet werden [54, 55]. Im WIR hat es bisher keine HIV-Infektion bei einem*r PrEP-Nutzer*in gegeben. Sind für die HIV-Prävention TasP und PrEP von großer Bedeutung, so dürfen 2 weitere Stellschrauben der Prävention nicht vernachlässigt werden: Aufklärungs- und Testkampagnen zur Verringerung der weiterhin hohen HIV-Spätdiagnosen und zur Steigerung der STI-Testraten und Therapien sowie das Kondom zum Schutz vor STI. Eine weitere Auswirkung der PrEP ist jedoch auch, dass STI bei PrEP-Nutzer*innen ansteigen, allerdings konzentrieren sich 80 % der STI auf 40 % der PrEP-Nutzenden [56]. Diese klar definierbaren Personen mit erhöhtem STI-Risiko werden trotz persönlicher Ansprache, Aufklärungskampagnen und Anleitung zu kontrolliertem Chemsex bisher nicht ausreichend erreicht.

Dass bei entsprechender Fokussierung auf eine Zielgruppe Präventionsbotschaften wirken, erkennt man an den HIV-Kampagnen: Selbst heute noch machen über 25-Jährige häufiger HIV-Tests als STI-Tests (ORT), weil ungeachtet bestehender anderer STI-Risiken eine größere Gefährdung durch HIV vermutet wird [57]. TasP und PrEP bieten einen sehr hohen Schutz vor einer HIV-Infektion in sexuell aktiven Populationen, jedoch keinen Schutz vor anderen STI – für diese ist das Kondom weiterhin wichtig. Bei sexuell aktiven, nicht monogam lebenden Personen kann „Sex ohne Kondom“ als Indikator für ein Infektionsrisiko mit anderen STI gelten und sollte die Indikation für einen STI-Test sein [56]. Durch die Einbindung der PrEP-Nutzer*innen in die medizinische Versorgung, also in STI-Testung und Therapie – wenn möglich einschließlich der Partner*innen –, kann mittelfristig eine Verringerung der STI-Infektionen erreicht werden.

Die Aktualität der Daten dieser Arbeit ermöglicht Erkenntnisse zum Sexualverhalten in der Zeitspanne vor/während der SARS-CoV-2-Pandemie, ohne/mit Lockdown. Betrachtet man die Nutzung der PrEP, so sind die Zahlen der Nutzer*innen in der IA im April 2020 kurzzeitig eingebrochen, jedoch begann die Nutzung der PrEP ab Mai wieder. Neben den verringerten Nutzerzahlen im April war eine Änderung des Einnahmeschemas, von einer täglichen zu einer Einnahme nach Bedarf („on demand“) zu beobachten. Diese Änderungen der Nutzung werden in Studien bestätigt [27–29]. An den Monatsstatistiken des GA lässt sich z. B. der wellenförmige Verlauf der Pandemie über das Jahr 2020 ablesen. Mit steigenden SARS-CoV-2-Inzidenzen sinken die Besucher*innenzahlen und die Rate positiver STI-Tests steigt an. Dieser recht typische Verlauf findet sich in der IA nicht. Allen vorliegenden Daten (ORT, COWIR, PrEP, IA und GA im WIR) ist gemeinsam, dass Menschen wegen SARS-CoV‑2 ihre Sexualkontakte durchaus reduziert haben. In der COWIR- und PrEP-Studie wurden diese zwischen 50 % bereits in Pre‑L und 80 % in Ex‑L reduziert und in der PrEP-Studie verringerte sich die Partner*innenzahl in Ex‑L im Durchschnitt von 13 auf 8 (bei einer Spannweite von 0–50 Partner*innen). Dennoch stiegen die STI-Raten in der IA im Vergleich zu 2019 deutlich, sodass diese Reduzierungen der Sexualkontakte entweder nicht ausreichend waren, wie teils auch in Zentren anderer Länder beschrieben [31], und/oder bei den „vertrauten“ Sexualpartner*innen und deren Partner*innen STI-Cluster vorlagen, die in diesen sozialen Netzen zu höheren STI-Inzidenzen führten. Dieses Beispiel zeigt, dass spezielle Präventionsbotschaften auch für außergewöhnliche Situationen, wie bspw. in der SARS-CoV-2-Pandemie, angeboten werden müssen, da Sexualität natürlich auch in Ausnahmezeiten und -situationen ausgelebt wird [58, 59].

Die hohen Nutzer*innenzahlen des ORT von 507 pro Monat in K2 zeigen die große Akzeptanz des anonymen Tests. Sie bestätigt, dass Onlineangebote gerade von jungen Menschen gerne und selbstverständlich genutzt werden [60, 61]. Es ist zu erwägen, Online-Risikotests als einen Ansatz von Prävention mit Risikoeinschätzung und Wissensvermittlung in Kampagnen einzubinden, auch weil sie eingeschränkte Zugänge zu Beratung und Risikoeinschätzung kompensieren können – ob in Pandemiezeiten oder aufgrund individueller Lebensumstände.

## Limitierungen

Ein direkter Vergleich der Daten aller untersuchten Populationen war nicht möglich, da die Fragestellungen nicht identisch waren und zum Teil Vorabergebnisse (COWIR-, PrEP-Studie) analysiert wurden. Einschränkend könnte auch sein, dass bei einigen Fragen ggf. gesellschaftlich angepasste Antworten gegeben wurden.

## Fazit

Neben allen oben genannten Erkenntnissen für Präventionsbotschaften gilt: Sexuelle Bildung sollte früh in der Schule und zu Hause beginnen, selbstverständlich sein für Bildende und zu Bildende, damit bereits Heranwachsende verantwortungsbewusst mit Sexualität umgehen können. Ein weiterer Fokus sollte auf der Versorgung liegen: Nur wenn Versorger*innen geschult sind, Risikokonstellationen wahrzunehmen und anzusprechen (Ausbildung Sexualmedizin), können STI durch Vorsorge und Screening besser erkannt werden. Dies erfordert eine Implementierung im Gesundheitssystem.

## Footnotes

1 Unter dem übergeordneten Begriff *Geschlecht* sind in diesem Kontext sowohl die geschlechtliche Identität im biologischen Sinne als auch Genderaspekte im Sinne gesellschaftlicher Geschlechterrollen gefasst.

2 Chemsex beschreibt die Nutzung der Substanzen Gammahydroxybuttersäure/Gamma-Butyrolacton (GHB/GBL, auch unter den Namen G, G‑Juice, Limo, Liquid oder Liquid Ecstasy bekannt), Mephedron, Ketamin, Crystal Meth und andere zum Sex.

3 Faustverkehr; bei dieser Praktik sind Verletzungsgefahr und HIV-/Hepatitis-C-Viren-Transmissionsrisiko erhöht.

4 Es bestehen hierbei Verletzungsrisiken der Schleimhaut.

5 Dieser führt u. a. zu fehlendem Schmerzempfinden und Kontrollverlust.

6 Die Autor*innen sehen zwischen den Kalenderjahren 2019 und 2020 einen relevanten Anstieg von verschiedenen STI, wobei es sich um Beobachtungen ohne statistische Auswertung handelt.

7 Hinsichtlich der Nachweisgrenze gilt die n = n-Formel: nicht nachweisbar = nicht übertragbar.
